# Genetic association between *TRAIL-R1* Thr209Arg and cancer susceptibility

**DOI:** 10.1038/srep10382

**Published:** 2015-08-28

**Authors:** Peiliang Geng, Jianjun Li, Ning Wang, Yunmei Liao, Juanjuan Ou, Rina Sa, Ganfeng Xie, Chen Liu, Hongtao Li, Lisha Xiang, Houjie Liang

**Affiliations:** 1 Department of Oncology and Southwest Cancer Center, Southwest Hospital Third Military Medical University, 29 Gaotanyan Main Street, Chongqing 400038, China

## Abstract

We aimed to determine the indecisive association between tumor necrosis factor-related apoptosis-inducing ligand receptor 1 (*TRAIL-R1*) Thr209Arg polymorphism and inherited susceptibility to cancer. A meta-analysis combining data on 9,517 individuals was performed to assess the association between *TRAIL-R1* Thr209Arg and cancer incidence. The summary ORs with 95% CI calculated with the fixed effects model suggested that Thr209Arg was not significantly associated with cancer susceptibility (homozygous model: OR 0.98, 95% CI 0.88–1.09; heterozygous model: OR 0.95, 95% CI 0.87–1.04; allele frequency model: OR 0.99, 95% CI 0.94–1.05; dominant model: OR 0.98, 95% CI 0.91–1.05; recessive model: OR 1.01, 95% CI 0.92–1.10). Stratified analysis by ethnicity and cancer type yielded similar null associations. These statistical data suggest that Thr209Arg in exon 4 of the *TRAIL-R1* gene may not represent a modifier of susceptibility to cancer.

Among the various genomic abnormalities, allelic loss at human chromosome 8p21 is particularly frequent in all kinds of cancer and thus has received widespread attention in recent years^1^,^2^. Tumor necrosis factor-related apoptosis-inducing ligand (TRAIL) is a homotrimeric cytokine located at chromosome band 8p21. It has been suggested that TRAIL is a promising anticancer agent due to its critical regulatory role in apoptosis, a cell suicide mechanism with an important role in maintaining normal cell cycling and abrogating the unwanted or potentially threatening cells^3^,^4^. TRAIL binds to the TRAIL receptor 1 (*TRAIL-R1*), a gene also known as *DR4* and *TNFRSF10A*^5^. *TRAIL-R1* enables cell death and triggers apoptotic proteases to regulate apoptosis through inducing the oligomerization of intracellular death domains required for the apoptotic signal transduction and forming an extracellular cysteine-rich, ligand-binding domain^6^,^7^,^8^,^9^.

The polymorphic *TRAIL-R1* encodes nearly 480 amino acids. Downregulation of *TRAIL-R1* may accelerate tumor formation and progression. Previous work has reported a significant relevance of lowly expressed *TRAIL-R1* to a variety of cancers and breast cancer cell lines^9^,^10^. The *TRAIL-R1* mutation is a frequent event that has been associated with many types of human malignancy^11^,^12^. There are multiple well-characterized polymorphisms in the *TRAIL-R1* gene, but the most extensively studied polymorphism has been the C > G substitution resulting in a threonine to arginine amino acid change in exon 4 (Thr209Arg, rs20575). Thr209Arg is of special interest in recent decade most likely due to the involvement in receptor ligand binding activity and stimulation of apoptotic pathways^12^. A great deal of attention has been directed to the testing of a hypothesis that Thr209Arg may modulate host susceptibility to cancer. However, the previous investigations, either in the form of genetic association study or meta-analysis, fail to provide compelling evidence^13^,^14^,^15^,^16^. The relatively small sample size may account a large part for the limited statistical power of these studies.

To determine whether Thr209Arg in the ectodomain of the *TRAIL-R1* gene is independently associated with cancer, we conducted a meta-analysis where all usable data identified through several medicine-specific databases have been incorporated.

## Materials and Methods

### Search strategy, inclusion criteria and data extraction

Using the combinations of polymorphism, polymorphisms, variants, genotypes, TRAIL receptor 1, *DR4*, and cancer, we searched the PubMed database (http://www.ncbi.nlm.nih.gov/pubmed), in an effort to identify all relevant peer-reviewed literature published prior to February, 2014. To get additional usable data, we screened all articles assessing the effects of Thr209Arg on cancer and their references. A study was considered eligible if the association between Thr209Arg and cancer susceptibility was investigated, the study subjects were composed of cancer patients and well-matched healthy controls, and count of Thr/Thr, Thr/Arg and Arg/Arg genotypes was clearly reported or information on genotype distribution was sufficiently provided in the research article. For the studies containing overlapped samples, the largest study with complete data was considered in the meta-analysis.

We collected data on authors, publication year, type of cancer, genotyping methods, study country, ethnicity of each population included, and genotype frequency for each of the eligible studies. To maximize data accuracy, the information listed above was extracted independently by two investigators.

### Statistical analysis

Based on genotypic and allelic data, we estimated cancer susceptibility (OR and 95% CI: odds ratio and 95% confidence interval) in relation to Thr209Arg for homozygous model, heterozygous model, allele frequency model, dominant model, and recessive model, by applying a fixed or random effects meta-analysis. Stratified analysis was conducted by ethnicity, cancer type and Hardy-Weinberg equilibrium (HWE), to assess the association for each subgroup.

Heterogeneity across studies was checked by the χ^2^-based Q-test^17^, to determine whether the Mantel-Haenszel method (fixed effects model, FEM)^18^ or the DerSimonian and Laird method (random effects model, REM)^19^ was used to pool the data from the published studies. In case of absence of inter-study heterogeneity (P values >0.05), we chose the former method; otherwise, the latter method was applied for pooling purpose.

HWE was checked for the control group in each study using χ^2^ test^20^. Sensitivity analysis was performed by consecutively excluding every study to see whether the single data set had obvious influence on the combined ORs. The Egger regression test and Begg’s funnel plots were utilized to determine publication bias^21^.

Meta-analysis was performed using the software STATA 12.0 (Stata Corporation, College Station, TX, USA). A P value <0.05 was deemed statistically significant.

## Results

### Summary of study characteristics

**Figure 1:**
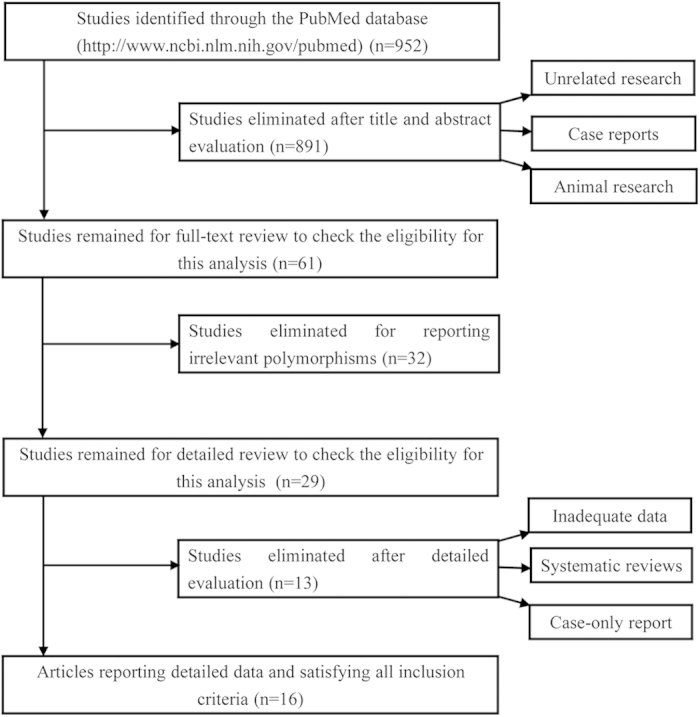
Flow diagram of study selection for meta-analysis.

We retrieved a total of 952 records matching pre-listed keywords. Title and abstract evaluation led to an elimination of 891 records. We then read the full text of all 61 articles and found 32 articles reported an association unrelated to the polymorphism being examined, 8 articles offered insufficient raw data, 4 articles were systematic reviews and 1 was case-only designed. After discarding all useless records, we at last included 16 articles^13^,^14^,^15^,^22^,^23^,^24^,^25^,^26^,^27^,^28^,^29^,^30^,^31^,^32^,^33^,^34^ (Fig. 1). Genotype and allele frequencies, along with main characteristics of the studies involved in this meta-analysis are detailed in Table 1. According to Table 1, breast cancer was the most studied cancer type, followed by lung cancer. Other cancers, such as cancers of bladder, hematological, gastric, ovarian, colorectal, liver and gallbladder were relatively less investigated and thereby merged into “other” category when performing meta-analysis. Caucasian and Asian ethnicities were all investigated, with Caucasian individuals outnumbering Asians. The genotype frequencies of Kuraoka *et al.* (2005) and Mittal *et al.* (2011) in control population were not in HWE, according to χ^2^ test.

### Meta-analysis

**Figure 2:**
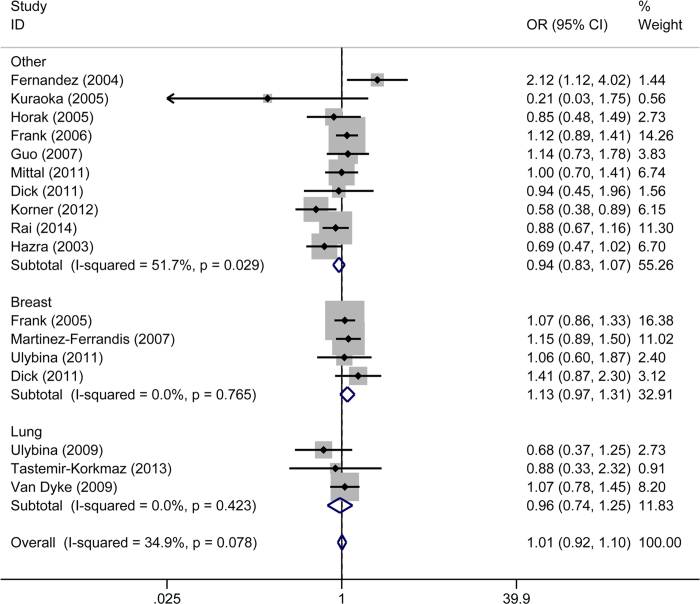
Meta-analysis using a fixed effects model for the association between cancer susceptibility and *TRAIL-R1* Thr209Arg stratified by ethnicity (recessive model). OR: odds ratio; CI: confidence interval; I-squared: measure to quantify the degree of heterogeneity in meta-analyses.

As shown in Table 2, there was no substantial inter-study heterogeneity and we hence selected the FEM for the calculation of pooled ORs. A fixed effects meta-analysis revealed that there was no overall association between Thr209Arg and cancer (homozygous model: OR 0.98, 95% CI 0.88–1.09; heterozygous model: OR 0.95, 95% CI 0.87–1.04; allele frequency model: OR 0.99, 95% CI 0.94–1.05; dominant model: OR 0.98, 95% CI 0.91–1.05; recessive model: OR 1.01, 95% CI 0.92–1.10, Fig. 2).

Similar results were seen when the data were stratified by ethnicity (Fig. 2), cancer type, and HWE deviation (Table 2).

**Figure 3:**
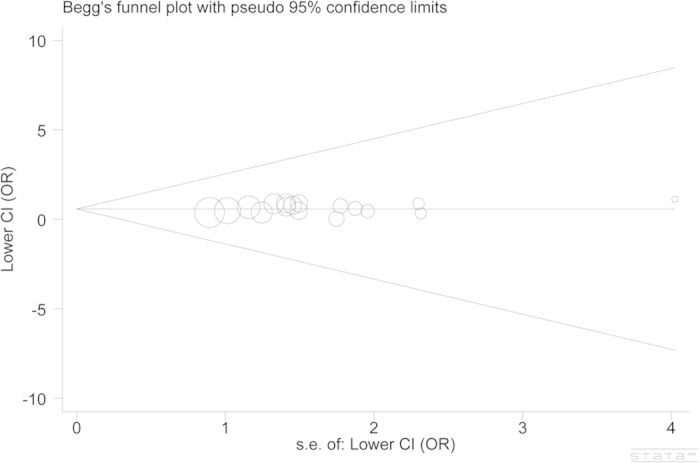
Begg’s funnel plot of publication bias test (recessive model). Each point represents a separate study for the indicated association. Log (OR): natural logarithm of OR; horizontal line: mean effect size.

With the aid of sensitivity analysis, we found that the combined effects remained stable when excluding each study. Neither did we find any evidence of significant publication bias, by using the funnel plots and Egger’s test (the recessive model: P = 0.304, Fig. 3).

## Discussion

Apoptosis is a defence mechanism against the malignant progression of cancer. Resistance to apoptosis destroys the balance between cell death and growth, thus facilitating tumorigenesis. *TRAIL-R1* is a transmembrane protein with a death domain essential for apoptotic regulation. Variations in this gene are proved detrimental, as these alternations suppress cell death and promote proliferation, two causes reported to account for increases in the likelihood of carcinogenesis^35^,^36^,^37^. A large body of research has focused on the role of *TRAIL-R1* Thr209Arg polymorphism in predisposition to cancer. However, there is a lack of consistency in the reported results. Hazra *et al.* conducted a large-scale study linking Thr209Arg with bladder cancer, providing epidemiological data that Thr209Arg plays a major role in the development of bladder cancer^22^. A subsequent study of German samples reported a decreased susceptibility of hematological malignant diseases in relation to *TRAIL-R1* polymorphic alleles^23^. Inconsistent with the former Germany study, Frank *et al.* genotyped 521 breast cancer cases and 1,100 control subjects and found an almost 4-fold increased susceptibility attributable to the carriage of 626Thr-683Ala haplotype, though Thr209Arg alone was not found to contribute towards incident breast cancer^24^. Similarly, the three most recent studies revealed substantially different findings, with Körner *et al.* and Rai *et al.* reporting 626Thr as an independent susceptibility factor for liver cancer^13^,^15^, and no associations between Thr209Arg and susceptibility to lung cancer, according to Taştemir-Korkmaz *et al.*^14^. The heterogeneity of the findings among investigations addressing the association of Thr209Arg polymorphism with cancer is biologically possible, as the etiology may vary widely due to the differences in cancer type. Another plausible explanation is related to the limited number of subjects in each published study. We here infer that the polymorphism being investigated may exert similar effects on all cancer types, and a sufficiently large study is needed to test this inference.

To provide compelling evidence of the association between Thr209Arg and cancer susceptibility, we performed a meta-analysis on 4,673 cancer cases and 4,844 controls from a total of 16 publications. Overall analysis revealed that this polymorphism predisposed no host susceptibility to cancer. We then performed stratified analysis by ethnicity, cancer type and HWE deviation to estimate the association for each subgroup, failing to demonstrate any statistical evidence of a significant association related to Thr209Arg. Our observations are not in accordance with those reported in a previous study, in which the investigators included 2,941 cases and 3,358 controls and found a marginal association (OR = 0.77, 95% CI:0.65–0.91; OR =0.84, 95% CI:0.72–0.99)^16^. The null associations implicated in the current analysis where nine additional studies have been included highlights the importance of a sufficient sample in detecting the true polymorphism-cancer associations. Despite the wide discrepancy in sample size between the two meta-analyses, we cannot rule out one possibility that Thr209Arg is a low-penetrance polymorphism and its effects on overall cancer and specific subtypes merit further investigations.

It is reported that genetic alterations in the *TRAIL-R1* gene lead to an impaired apoptotic mechanism, one of the prerequisites required for the development of cancer^11^,^12^. Functional studies have exhibited data on increased possibility to develop cancers of head and neck, lung, and gastric attributed to the nucleotide substitution in ectodomain of *TRAIL-R1*^12^. Several lines of evidence from candidate gene studies lend further support to the notion that *TRAIL-R1* Thr209Arg represents an effect modifier for cancer. 209Arg/Arg genotype was shown to modulate bladder cancer susceptibility via mediating the capacity of receptor ligand complexes involved in apoptotic pathways^23^. In addition, the 209Thr allele modifies risk of breast cancer by regulating TRAIL binding efficiency^24^. According to these data, we hypothesize that Thr209Arg may confer host susceptibility to cancer. This hypothesis nevertheless remains to be tested.

We have to address the limitations of this analysis. First, most of the single studies included have a small number of individuals, making the total sample underpowered to detect the association between Thr209Arg and various types of cancer. Second, it is important to note that the vast majority of published studies employed samples of Caucasian ancestry, thus the estimation of cancer susceptibility in Asians may be derived by chance as a result of sample insufficiency. Third, similar to many meta-analyses, we categorized the populations into either Caucasian or Asian ethnicity, which may lead to overgeneralization in results. For example, although Thr209Arg does not modify cancer susceptibility in total Asian populations, but it may have effects on some specific populations, such as Chinese and Japanese. The above-mentioned shortcomings suggest the necessity of further studies.

To sum up, our meta-analysis indicated that *TRAIL-R1* Thr209Arg polymorphism was not significantly associated with overall cancer susceptibility. Stratified analysis by ethnicity and cancer type yielded similar results. The present findings, along with those suggested in previous analyses, merit further investigation involving more cancer types and ethnic groups.

## Additional Information

**How to cite this article**: Geng, P. *et al.* Genetic association between *TRAIL-R1* Thr209Arg and cancer susceptibility. *Sci. Rep.* **5**, 10382; doi: 10.1038/srep10382 (2015).

## Figures and Tables

**Table 1:** Characteristics of studies involved in this meta-analysis.

First author(year)	Country	Ethnicity	Cancertype	Genotypingmethods	Cases		Controls		HWE
					Thr/Thr-Arg/Thr-Arg/Arg	Thr-Arg	Thr/Thr-Arg/Thr-Arg/Arg	Thr-Arg	
Hazra (2003)	America	Caucasian	Bladder	PCR-RFLP	191-62	/	139-76	/	/
Fernandez (2004)	Spain	Caucasian	Hematological	PCR-RFLP	46-23-41	115-105	26-49-16	101-81	Y
Frank (2005)	Germany	Caucasian	Breast	Taqman	120-242-157	482-556	222-566-312	1010-1190	Y
Kuraoka (2005)	Japan	Asian	Gastric	PCR-RFLP	250-23-1	523-25	317-21-6	655-33	N
Horak (2005)	Austria	Caucasian	Ovarian	PCR-RFLP	13-51-28	77-107	14-50-36	78-122	Y
Frank (2006)	Germany	Caucasian	Colorectal	Taqman	156-286-208	598-702	118-298-167	534-632	Y
Martinez-Ferrandis (2007)	Spain	Caucasian	Breast	Taqman	110-257-163	477-583	107-244-128	458-500	Y
Guo (2007)	China	Asian	Gastric	PCR-RFLP	30-55-50	115-155	42-72-55	156-182	Y
Ulybina (2009)	Russia	Caucasian	Lung	AS-PCR	28-61-22	117-105	29-49-32	107-113	Y
Van Dyke (2009)	American	Caucasian	Lung	PCR-RFLP	258-104	/	274-101	/	/
Mittal (2011)	India	Asian	Bladder	PCR-RFLP	17-97-86	131-269	15-113-97	143-307	N
Ulybina (2011)	Russia	Caucasian	Breast	PCR-RFLP	33-61-27	127-115	37-75-30	149-135	Y
Dick (2011)	Germany	Caucasian	Breast	Taqman	196-279-132	671-543	43-78-22	164-122	Y
Dick (2011)	Germany	Caucasian	Ovarian	Taqman	27-50-13	104-76	43-78-22	164-122	Y
Korner (2012)	Germany	Caucasian	Liver	PCR-RFLP	40-87-32	167-151	62-166-121	290-408	Y
Tastemir-Korkmaz (2013)	Turkey	Caucasian	Lung	PCR-RFLP	12-34-14	58-62	10-12-8	32-28	Y
Rai (2014)	India	Asian	Gallbladder	AS-PCR	36-181-183	253-547	17-101-128	135-357	Y

**Table 2:** Summary ORs (95% CI) for TRAIL-R1 Thr209Arg and cancer.

Thr209Arg	N*	Arg/Arg vs. Thr/Thr		Arg/Thr vs. Thr/Thr		Arg vs. Thr		Arg/Arg + Arg/Thr vs.Thr/Thr		Arg/Arg vs. Arg/Thr +Thr/Thr	
		Homozygous model		Heterozygous model		Allele frequency model		Dominant model		Recessive model	
		OR (95%CI)	P_H_	OR (95%CI)	P_H_	OR (95%CI)	P_H_	OR (95%CI)	P_H_	OR (95%CI)	P_H_
Cancer type											
Breast	4	1.04 (0.88, 1.22)	0.871	0.95 (0.84, 1.09)	0.948	1.02 (0.94, 1.11)	0.925	0.98 (0.88, 1.09)	0.973	1.13 (0.97, 1.31)	0.765
Lung	3	0.93 (0.53, 1.62)	0.563	1.15 (0.76, 1.75)	0.659	0.97 (0.73, 1.28)	0.568	1.06 (0.75, 1.50)	0.679	0.96 (0.74, 1.25)	0.423
Other	10	0.94 (0.81, 1.09)	0.768	0.94 (0.83, 1.06)	0.651	0.97 (0.90, 1.04)	0.906	0.96 (0.88, 1.06)	0.996	0.94 (0.83, 1.07)	0.029
											
Ethnicity											
Caucasian	13	0.99 (0.87, 1.12)	0.931	0.94 (0.85, 1.03)	0.822	0.99 (0.93, 1.06)	0.928	0.97 (0.90, 1.05)	0.999	1.02 (0.93, 1.13)	0.049
Asian	4	0.96 (0.77, 1.19)	0.525	1.02 (0.83, 1.25)	0.803	0.98 (0.87, 1.10)	0.899	1.00 (0.86, 1.16)	0.971	0.94 (0.78, 1.14)	0.397
											
HWE											
Y	15	0.99 (0.88, 1.10)	0.970	0.95 (0.86, 1.04)	0.912	0.99 (0.93, 1.05)	0.958	0.97 (0.91, 1.05)	1.000	1.01 (0.92, 1.11)	0.070
N	2	0.89 (0.61, 1.31)	0.171	1.06 (0.77, 1.45)	0.350	0.98 (0.81, 1.19)	0.903	1.00 (0.78, 1.29)	0.689	0.94 (0.67, 1.32)	0.153
All	17	0.98 (0.88, 1.09)	0.949	0.95 (0.87, 1.04)	0.919	0.99 (0.94, 1.05)	0.985	0.98 (0.91, 1.05)	1.000	1.01 (0.92, 1.10)	0.078

^*^number os studies.

## References

[b1] Fujiwara Y. *et al.* A 3-Mb physical map of the chromosome region 8p21.3-p22, including a 600-kb region commonly deleted in human hepatocellular carcinoma, colorectal cancer, and non-small cell lung cancer. Genes Chromosomes Cancer 10, 7–14 (1994). 7519877 10.1002/gcc.2870100103

[b2] El-Naggar A. K. *et al.* Localization of chromosome 8p regions involved in early tumorigenesis of oral and laryngeal squamous carcinoma. Oncogene 16, 2983–7 (1998). 9662330 10.1038/sj.onc.1201808

[b3] Ashkenazi A. & Dixit V. M. Death receptors: signaling and modulation. Science 281, 1305–8 (1998). 9721089 10.1126/science.281.5381.1305

[b4] Walczak H. *et al.* Tumoricidal activity of tumor necrosis factor-related apoptosis-inducing ligand *in vivo*. Nat. Med. 5, 157–63 (1999). 9930862 10.1038/5517

[b5] Pan G. *et al.* The receptor for the cytotoxic ligand TRAIL. Science 276, 111–3 (1997). 9082980 10.1126/science.276.5309.111

[b6] Sheridan J. P. *et al.* Control of TRAIL-induced apoptosis by a family of signaling and decoy receptors. Science 277, 818–21 (1997). 9242611 10.1126/science.277.5327.818

[b7] Hymowitz S. G. *et al.* Triggering cell death: the crystal structure of Apo2L/TRAIL in a complex with death receptor 5. Mol. Cell 4, 563–71 (1999). 10549288 10.1016/s1097-2765(00)80207-5

[b8] MacFarlane M. *et al.* Mechanisms of resistance to TRAIL-induced apoptosis in primary B cell chronic lymphocytic leukaemia. Oncogene 21, 6809–18 (2002). 12360407 10.1038/sj.onc.1205853

[b9] Seitz S. *et al.* Mutation analysis and mRNA expression of trail-receptors in human breast cancer. Int. J. Cancer 102, 117–28 (2002). 12385006 10.1002/ijc.10694

[b10] Shin M. S. *et al.* Mutations of tumor necrosis factor-related apoptosis-inducing ligand receptor 1 (TRAIL-R1) and receptor 2 (TRAIL-R2) genes in metastatic breast cancers. Cancer Res. 61, 4942–6 (2001). 11431320

[b11] Lee S. H. *et al.* Somatic mutations of TRAIL-receptor 1 and TRAIL-receptor 2 genes in non-Hodgkin’s lymphoma. Oncogene 20, 399–403 (2001). 11313970 10.1038/sj.onc.1204103

[b12] Fisher M. J. *et al.* Nucleotide substitution in the ectodomain of trail receptor DR4 is associated with lung cancer and head and neck cancer. Clin. Cancer Res. 7, 1688–97 (2001). 11410508

[b13] Korner C. *et al.* TRAIL receptor I (DR4) polymorphisms C626G and A683C are associated with an increased susceptibility for hepatocellular carcinoma (HCC) in HCV-infected patients. BMC Cancer 12, 85 (2012). 22401174 10.1186/1471-2407-12-85 PMC3372437

[b14] Tastemir-Korkmaz D., Demirhan O., Kuleci S. & Hasturk S. There is no significant association between death receptor 4 (DR4) gene polymorphisms and lung cancer in Turkish population. Pathol. Oncol. Res. 19, 779–84 (2013). 23661154 10.1007/s12253-013-9643-z

[b15] Rai R. *et al.* Death receptor (DR4) haplotypes are associated with increased susceptibility of gallbladder carcinoma in north Indian population. PLoS One 9, e90264 (2014). 24587306 10.1371/journal.pone.0090264 PMC3938657

[b16] Chen B. *et al.* TRAIL-R1 polymorphisms and cancer susceptibility: an evidence-based meta-analysis. Eur. J. Cancer 45, 2598–605 (2009). 19643596 10.1016/j.ejca.2009.06.023

[b17] Zintzaras E. & Ioannidis J. P. Heterogeneity testing in meta-analysis of genome searches. Genet Epidemiol 28, 123–37 (2005). 15593093 10.1002/gepi.20048

[b18] Mantel N. & Haenszel W. Statistical aspects of the analysis of data from retrospective studies of disease. J Natl Cancer Inst 22, 719–48 (1959). 13655060

[b19] DerSimonian R. & Laird N. Meta-analysis in clinical trials. Control Clin Trials 7, 177–88 (1986). 3802833 10.1016/0197-2456(86)90046-2

[b20] Zintzaras E. & Lau J. Synthesis of genetic association studies for pertinent gene-disease associations requires appropriate methodological and statistical approaches. J Clin Epidemiol 61, 634–45 (2008). 18538260 10.1016/j.jclinepi.2007.12.011

[b21] Egger M., Davey Smith G., Schneider M. & Minder C. Bias in meta-analysis detected by a simple, graphical test. BMJ 315, 629–34 (1997). 9310563 10.1136/bmj.315.7109.629 PMC2127453

[b22] Hazra A. *et al.* Death receptor 4 and bladder cancer susceptibility. Cancer Res 63, 1157–9 (2003). 12649168

[b23] Fernandez V. *et al.* Frequent polymorphic changes but not mutations of TRAIL receptors DR4 and DR5 in mantle cell lymphoma and other B-cell lymphoid neoplasms. Haematologica 89, 1322–31 (2004). 15531454

[b24] Frank B. *et al.* Association of death receptor 4 haplotype 626C-683C with an increased breast cancer susceptibility. Carcinogenesis 26, 1975–7 (2005). 15975957 10.1093/carcin/bgi164

[b25] Kuraoka K. *et al.* A single nucleotide polymorphism in the extracellular domain of TRAIL receptor DR4 at nucleotide 626 in gastric cancer patients in Japan. Oncol Rep 14, 465–70 (2005). 16012731

[b26] Horak P. *et al.* Common death receptor 4 (DR4) polymorphisms do not predispose to ovarian cancer. Gynecol Oncol 97, 514–8 (2005). 15863153 10.1016/j.ygyno.2005.01.021

[b27] Frank B. *et al.* Death receptor 4 variants and colorectal cancer susceptibility. Cancer Epidemiol Biomarkers Prev 15, 2002–5 (2006). 17035413 10.1158/1055-9965.EPI-06-0053

[b28] Martinez-Ferrandis J. I. *et al.* Polymorphisms in TRAIL receptor genes and susceptibility of breast cancer in Spanish women. Cancer Biomark 3, 89–93 (2007). 17522430 10.3233/cbm-2007-3203

[b29] Guo L., Xia B., Song Q. & Li C. Association of DR4 gene polymorphism in Chinese patients with gastroduodenal diseases. Med J Wuhan Univ. 28, 93–95 (2007).

[b30] Ulybina Y. M. *et al.* Coding polymorphisms in Casp5, Casp8 and DR4 genes may play a role in predisposition to lung cancer. Cancer Lett 278, 183–91 (2009). 19203830 10.1016/j.canlet.2009.01.012

[b31] Van Dyke A. L. *et al.* Cytokine and cytokine receptor single-nucleotide polymorphisms predict susceptibility for non-small cell lung cancer among women. Cancer Epidemiol Biomarkers Prev 18, 1829–40 (2009). 19505916 10.1158/1055-9965.EPI-08-0962 PMC3771080

[b32] Mittal R. D. *et al.* Association of death receptor 4, Caspase 3 and 5 gene polymorphism with increased susceptibility to bladder cancer in North Indians. Eur J Surg Oncol 37, 727–33 (2011). 21700414 10.1016/j.ejso.2011.05.013

[b33] Ulybina Y. M. *et al.* Distribution of coding apoptotic gene polymorphisms in women with extreme phenotypes of breast cancer predisposition and tolerance. Tumori 97, 248–51 (2011). 21617726 10.1177/030089161109700222

[b34] Dick M. G. *et al.* Association of death receptor 4 variant (683A > C) with ovarian cancer susceptibility in BRCA1 mutation carriers. Int J Cancer 130, 1314–8 (2012). 21484799 10.1002/ijc.26134

[b35] Evan G. I. & Vousden K. H. Proliferation, cell cycle and apoptosis in cancer. Nature 411, 342–8 (2001). 11357141 10.1038/35077213

[b36] Koornstra J. J. *et al.* Expression of tumour necrosis factor-related apoptosis-inducing ligand death receptors in sporadic and hereditary colorectal tumours: potential targets for apoptosis induction. Eur J Cancer 41, 1195–202 (2005). 15911244 10.1016/j.ejca.2005.02.018

[b37] Bouralexis S., Findlay D. M. & Evdokiou A. Death to the bad guys: targeting cancer via Apo2L/TRAIL. Apoptosis 10, 35–51 (2005). 15711921 10.1007/s10495-005-6060-0

